# Dilated Cardiomyopathy – Exploring the Underlying Causes

**DOI:** 10.18103/mra.v12i12.6111

**Published:** 2024-12-24

**Authors:** David F. Wieczorek

**Affiliations:** Department of Molecular and Cellular Biosciences, University of Cincinnati College of Medicine, 231 Albert Sabin Way

## Abstract

Cardiovascular disease is one of the world’s leading causes of natural mortality, taking approximately 18 million lives each year. Dilated cardiomyopathy, a subgroup of cardiac diseases, has an annual incidence of 5 – 8 cases per 100,000 for European and North American populations. Common features of dilated cardiomyopathy include cardiac chamber enlargement, impaired systolic function, reduced ejection fraction, and arrhythmias, with an endpoint of ventricular dilation and heart failure. The focus of this paper is to review the non-genetic and genetic etiologies that lead to dilated cardiomyopathy. The non-genetic causes of dilated cardiomyopathy that are discussed include viruses, cardiotoxicity, recreational drugs, and chemotherapeutic medications. For the genes that lead to dilated cardiomyopathy, the focus of this paper is on cytoskeletal and sarcomeric protein genes. Our scope in defining this area will be to explore numerous mouse models that incorporate mutations found in humans that lead to dilated cardiomyopathy. The purpose of the paper is to define the morphological and physiological consequences of these mutations and how this information has furthered our understanding of the disease. Having gained invaluable knowledge from these animal models, it is hoped that new and improved therapeutic approaches can be developed for the treatment and prevention of dilated cardiomyopathy.

## Introduction

Cardiovascular disease is one of the world’s leading causes of natural mortality, taking approximately 18 million lives each year. As a group, they include disorders of the heart and blood vessels that include coronary heart disease, cerebrovascular disease, hypertrophic and dilated cardiomyopathies, and other conditions that often lead to heart failure. Heart failure, the progressive loss of cardiac function affects an estimated 6.5 million Americans and 26 million people worldwide, with medical costs between $40 – 60 billion per year.

Cardiomyopathies are a heterogenous group of diseases, defined by a pathologically abnormal myocardium. These diseases include dilated (DCM), hypertrophic (HCM), restrictive (RCM) and arrhythmogenic right ventricular (ARVC) cardiomyopathies. In addition, there are also non-classified cardiomyopathies that frequently present as a syndrome of heart failure (HF)^1^. Global estimates for the prevalence of cardiomyopathy are 2.5 million cases with DCM being a significant cause of cardiac arrest^2^. The annual incidence is 5 – 8 cases per 100,000 for European and North American populations. Overall, about 1/250 people will develop DCM and 1/500 will develop HCM^3^. In the UK, DCM is responsible for 20% of all heart failure diagnoses^4^, with the estimated cost of DCM patient care being $4–10 billion/year in the United States^5^.

DCM is typically defined as the presence of left ventricular or biventricular dilation with systolic dysfunction in the absence of abnormal loading conditions or significant coronary artery disease^2,3^. It is often diagnosed through *in vivo* imaging with either echocardiographic or magnetic resonance imaging (MRI). The most common feature is enlargement of the heart which often affects all four cardiac chambers, especially late in the disease process. Early in the disease process, there may be structural cardiac dilation with a minimally reduced function. However, if the DCM has progressed to heart failure, the cardiac ejection fraction may be less than 40%, with some patients exhibiting a left ventricular ejection fraction of 24%^1^. Although DCM is often characterized by symptomatic heart failure, it can also present with arrhythmias and thromboembolic conditions. Despite significant medical advances in diagnosis and treatment, DCM is still associated with significant mortality and survival is approximately 60% after 10 years^6,7^.

## Etiologies of Dilated Cardiomyopathy: Non-Genetic Causes and Associations

Several non-genetic causes have been established which can lead to DCM. These include infections, autoimmunity, toxins (i.e. alcohol, recreational drugs, cancer therapy), endocrinopathies, and tachyarrhythmias^3^. Human and animal studies have demonstrated that some of these stressful agents initially result in myocarditis, with DCM and heart failure occurring afterwards^8,9^. However, spontaneous resolution of acute myocarditis with recovery of cardiac function is common (40% - 60%), and the subsequent development of DCM being more variable (14% - 52%)^10,11^. The progression of myocarditis to overt DCM may also not occur for several years, which may complicate potential therapies.

Several viruses have been implicated to play a role in the development of DCM and heart failure. These viruses include enterovirus, herpes viruses, parvovirus B 19, Epstein-Barr virus, Hepatitis C virus, and Covid-19 among others^1,2^. Human herpesvirus, parvovirus B19 and enteroviruses are the most common viral infectious agents leading to DCM. Viral infection can lead to direct cytotoxic effects, such as myocyte necrosis and activation of immune responses. Also, human immunodeficiency viral (HIV) infections that lead to a DCM condition have been noted to occur in sub-Saharan Africa.^1^ DCM can also result from a systemic immune-mediated disease process with inflammation and dysregulation of the immune system implicated in the pathogenesis of DCM^12–14^. Systemic immune-mediated conditions that may play a potential role in the development of DCM include rheumatoid arthritis, systemic sclerosis, and myasthenia gravis.

Autoinflammatory conditions that can act as a trigger for DCM include Crohn’s disease, ulcerative colitis and reactive arthritis. An increased activity of T lymphocytes which target viral and cellular components, such as heat shock, mitochondrial, and sarcomeric proteins, can occur. This inflammatory myocardial damage may contribute to altered systolic and diastolic function, eventually leading to DCM and HF. With SIDS (systemic immune-mediated diseases), autoantibodies may promote inflammatory responses or may directly cause cardiac damage through myocyte loss and fibrosis, with the resulting development of DCM^15^. It should be noted that with all of these potential agents whose end result leads to DCM and heart failure that a genetic predisposition may influence the onset and severity of this pathological phenotype.

Cardiotoxicity has long been known as a contributing factor in the development of DCM. Alcohol abuse and exposure to risky recreational drugs, such as amphetamines, cocaine, and anabolic steroids are causative agents in heart disease. With respect to alcohol abuse, alcoholism without abstinence is a strong predictor of cardiac death. In fact, studies show that patients with alcoholic cardiomyopathy without abstinence had significantly worse outcomes than patients with alcoholic cardiomyopathy and abstinence, or patients with idiopathic dilated cardiomyopathy^16^. Potential mechanisms involved in the toxic effects of alcohol on the myocardium include impaired mitochondrial oxidative function, altered myofilament protein synthesis, and dysregulation of cytosolic calcium which contributes to a decrease in myofilament Ca^2+^ sensitivity, a hallmark of DCM^17^.

Amphetamines are drugs that are structurally related to naturally occurring biogenic amines (dopamine, serotonin catecholamines). The precise mechanism of how amphetamine usage contributes to DCM is unclear, but may be related to tachycardia-induced cardiomyopathy or recurrent hypertensive crises leading to left ventricular failure. Related drugs, such as ephedra (Ma Huang) and ecstasy have been associated with stroke, myocardial infarction, arrhythmias and cardiomyopathy; ephedra appears to act by increasing catecholamines in synaptic areas of the brain and heart by increasing heart rate, blood pressure, cardiac output and peripheral resistance^17^. Cocaine is associated with multiple cardiovascular complications, including chest pain, myocardial ischemia/infarction, arrhythmias, and stroke. Cocaine blocks the reuptake of dopamine and epinephrine at the postsynaptic receptor, resulting in increased sympathetic activation, increased calcium flux, enhanced oxidative stress, and promotion of intracoronary thrombus formation; common pathological findings include sinus tachycardia with frequent arrhythmias, increased left ventricular hypertrophy and dysfunction^17^.

Chemotherapeutic drugs, such as anthracycline (doxorubicin), monoclonal antibodies, tyrosine kinase inhibitors, and immunomodulating agents have a history of leading to systolic dysfunction and heart failure. Tumors commonly treated with doxorubicin include breast and esophageal carcinomas, osteosarcoma, soft tissue sarcomas, and Hodgkin’s and non-Hodgkin’s lymphomas. The effects of these cardiotoxic agents may not manifest their pathological phenotype in the patient immediately and may take years to develop after a build-up of the drugs in the patient. Because of the cardiotoxic effects of doxorubicin, its usage has been curtailed. Also, since cancer patients often receive multiple drugs and/or radiotherapy, prediction of cardiovascular problems is complicated, but sometimes can be reversed following withdrawal of the offending agent(s). It should also be noted that with many of these cytotoxic agents, certain modifier genes may potentially dramatically aggravate or ameliorate the development of DCM and its pathology dependent upon the level of stress associated with the infections, toxins and other agents.

## Genes and Their Mutations That Lead to Dilated Cardiomyopathy

The genes associated with familial DCM are numerous and varied in their function. Approximately 20 – 40% of DCM cases are associated with a familial inheritance pattern^18^, and the associated genetic mutations often demonstrate autosomal dominant inheritance patterns. Over 50 genes have been identified that are linked to DCM, which encode proteins in the sarcomere, ion channels, cytoskeleton, nuclear envelope, and mitochondria^19,20^. The most common proteins associated with DCM are found in 2 major subgroups – cytoskeletal and sarcomeric. With respect to the cytoskeletal group, lamin A/C, desmin, dystrophin and sarcoglycans are the most common. The sarcomeric proteins that encode DCM inducing mutations include titin, α- and β-myosin heavy chain, myosin binding protein C, actin, tropomyosin, troponin T, I, and C, desmin, vinculin, and muscle LIM protein^21,22^. Mutations in Z-disk proteins (ZASP, muscle-LIM, actinin) and the sarcoplasmic reticulum protein phospholamban can also result in the DCM phenotype. Interestingly, many of the genes that can result in the DCM phenotype can also cause hypertrophic cardiomyopathy when encoding different mutations. Titin, lamin A/C and β-myosin heavy chain account for greater than 25% of genetically inherited DCM^22,23^. Titin, the longest human protein, is composed of 34,350 amino acids with a molecular weight of 3,816,030 Da. This sarcomeric protein anchors into the z-disc and functions as a scaffold for both thick and thin filament proteins in striated muscle and spans the entire sarcomere. Many of the DCM associated mutations in titin encode premature stop codons, resulting in truncated forms of the protein. These mutations and others often lead to sarcomere instability, decreased binding to the Z-disc, and a decreased stretch response during sarcomeric contraction. The mechanism associated with the development of DCM with sarcomeric protein-induced DCM are thought to be due to abnormalities in force generation. Mutations in myosin heavy chain may disrupt the actin-myosin interaction associated with force generation and cross-bridge movement of the myofilaments. Mutations in the troponin complex and tropomyosin may alter Ca^2+^ binding or the repositioning of tropomyosin to facilitate actin-myosin interactions. Mutations in tropomyosin that cause DCM often result in decreased tropomyosin flexibility, which impairs actin binding, decreases myofilament Ca^2+^ sensitivity, and impairs systolic and diastolic function^24^.

For mutated cytoskeletal proteins that cause DCM, defects in force transmission are thought to be responsible for the pathological phenotype. Mutations in the lamin A gene account for approximately 6% of all DCM mutations^25^. Lamin proteins are associated with intermediate filaments which support the nuclear membrane. Mutations in the associated protein lead to nuclear membrane damage and/or chromatin disorganization. In the heart, lamin A mutations cause dysrhythmias, including sinus node and AV node dysfunction, atrial and ventricular fibrillation, and sudden cardiac death.

The regulation of calcium and sodium in cardiomyocytes is paramount for the proper physiological functioning of the heart. Mutations in proteins associated with handling and transport of these ions can lead to aberrant systolic and diastolic function. Phospholamban, a regulator of the sarcoplasmic reticulum Ca^2+^- ATPase pump, has several autosomal dominant mutations that result in DCM. In fact, the R14del mutation in PLN is associated with a founder effect in the Netherlands which results in a severe DCM phenotype.^26^ However, as with many mutations, modifier genes may also play a role in decreasing the severe phenotype associated with this R14del mutation.

## Animal Models of Dilated Cardiomyopathy

### NATURALLY OCCURRING DILATED CARDIOMYOPATHY ANIMAL MODELS

There are two naturally occurring strains of hamsters that develop DCM: BIO14.6 and CHF 147^27,28^. The DCM phenotype develops following a prolonged period of cardiac dysfunction, with myolysis developing between 30 – 40 days, hypertrophy at 150 days, DCM at 250 days, and congestive heart failure at 1 year^29^. The progression to heart failure can be monitored by echocardiography.

## Genetically Modified Models of Dilated Cardiomyopathy

### MOUSE MODELS OF LAMIN A

Lamins are implicated in many different cellular processes, including regulation of gene expression, mechanosensing, DNA replication and nuclear to cytoplasmic transport. Mutations in the lamin A and C genes lead to a plethora of different diseases, which includes DCM with an incidence of 5 – 8%.^23^ These lamin mutations include missense, nonsense, splice site, and frameshift mutations and are usually inherited in an autosomal dominant fashion.

Lamin A mutations are frequently associated with dysrhythmias in the heart that include sinus node dysfunction, atrial fibrillation, AV node dysfunction, and sudden cardiac death, although arrhythmias and left ventricular dysfunction phenotypes are frequently not manifest in the mouse models^23^. The reason for the defects in the cardiac conduction systems for lamin A mutations is not well understood. Targeted deletion in mouse models of the lamin A gene show cardiomyocyte degeneration and DCM with conduction abnormalities by 4–6 weeks of age^30,31^. Isolated cardiomyocytes demonstrate ultrastructural changes with destabilization of nuclear lamina structure and enhanced nuclear deformability. Heterozygous male mice for lamin A mutations exhibit DCM later in life, with altered nuclear morphology and perinuclear desmin organization in their cardiomyocytes^32^. Interestingly, moderate exercise appears to modulate the DCM development in these mice, as did inhibition of the mTOR pathway by temsirolimus or rapamycin^33,34^.

### MOUSE MODELS OF TITIN

Mutations in both thick and thin sarcomeric proteins result in DCM. As previously mentioned, mutations in titin are one of the most prevalent causes of DCM. Titin variants in the gene’s highly conserved 363 exons are present in approximately one in five patients with DCM and one in 200 individuals in the general population^35^. Depending upon the location of these variants, the onset of pathogenicity varies often affecting sarcomere stability that can be disrupted by mechanical stress. Normally, titin is involved in directing assembly of contractile filaments, providing elasticity by its serial spring elements, and is thought to be involved in myofibrillar cell signaling.

In order to understand the molecular and pathological mechanisms associated with the development of this pathological condition associated with titin mutations, mouse models incorporating various DCM causing mutations have been generated. Investigators developed a knock-in mouse model that mimics a human DCM titin-based disease^36^. The human insertion mutation of 2 nucleotides in exon 326 of the titin gene leads to a translational reading frameshift that adds 4 additional amino acids prior to encountering a premature stop codon. In this model, mutant homozygous embryos of the titin knock-in mutation developed severe defects in sarcomere formation and died before E9.5^36,37^. Heterozygous mice were viable and demonstrated normal cardiac morphology and physiology. However, when subject to the pharmacological stressor angiotensin II or isoproterenol, they recapitulate the DCM phenotype, including impaired fractional shortening and diffuse myocardial fibrosis, which is similar to humans^36^. Also, when transverse aortic constriction (TAC) is performed on these heterozygous titin knock-in mice, they exhibit cardiac systolic abnormalities and fibrosis which is reflective of the human disease pathology^38^. It is not clear how the mutant truncated titin protein malfunctions or how it results in cardiac remodeling^39^; however, recent studies demonstrate that antisense-mediated exon skipping in human and mouse models of DCM carrying the frameshift mutation in titin exon 326 can improve myofibril assembly and function in homozygous embryos and rescue the DCM phenotype in heterozygous animals^40^.

### MOUSE MODELS OF MYOSIN HEAVY CHAIN

Mutations in myosin heavy chain are a common cause of inherited DCM^41^. There are approximately 50 variants that cause this pathological condition with most of them located in exons encoding the S1 region of the myosin head protein^42^. These variants include missense, splice donor, and in-frame deletion mutations. Patients with these mutations often exhibit significant cardiomyopathic symptoms, frequently with childhood presentation, and often associated with left ventricular noncompaction. However, ventricular arrhythmias were significantly lower compared with laminin-related or titin DCM patients^42^.

Two mouse models with mutations in myosin heavy chain that lead to the DCM phenotype have been engineered^43^. These two missense mutation models are: S532P and F764L in the alpha-cardiac myosin heavy chain. Each of these mutations maps to the S1 head region of myosin. The S532P mutation occurs within the actin-binding domain, and the F764L mutation occurs within the converter domain. These murine models exhibit the physiological, cellular, and molecular characteristics consistent with human DCM phenotypes. Contractile function of isolated cardiomyocytes was depressed along with reduced fractional shortening. Actin-activated ATPase is reduced in both mutants, as is *in vitro* sliding motility of actin. These results suggest that the depressed molecular function in cardiac myosin may lead to cardiac remodeling with an endpoint of DCM and heart failure. Interestingly, the development and analysis of a *Drosophila* model of the S532P mutation shows similar morphological and physiological effects of this mutation: reduced rates of actin-dependent ATPase activity, increased rate of actin detachment, decreased power output of flight muscle, and cardiac dilation that is gene dose-dependent^44^.

### MOUSE MODEL OF MYOSIN BINDING PROTEIN C

Myosin binding protein C (MyBP-C) is a large, abundant protein that participates in both myofibrillar structural and physiological functions. During cardiac development, its expression correlates with the onset of myofibrillogenesis. Functionally, MyBP-C contributes to contractile function by stimulating cardiac actomyosin ATPase or influencing myofibril tension generation and contractile velocity.^45^ A DCM mouse model was engineered encoding a truncated form of MyBP-C which altered the MHC-binding and titin-binding domains^45^. This truncated protein was 15kDa smaller than the 150 kDa wildtype MyBP-C protein due to the removal of exons 29 – 31 from the targeted gene, which reflects a human DCM mutation. Homozygous mice bearing the mutated MyBP-C alleles are viable, but exhibit neonatal onset of a progressive DCM with cardiomyocyte hypertrophy, myofibrillar disarray, fibrosis, left ventricular dilation and contractile dysfunction. Additional studies on these mice demonstrate an upregulation of inflammatory pathways in the DCM hearts which likely occurs in response to cellular damage triggered by the MyBP-C mutation and contractile dysfunction^46^.

### MOUSE MODELS OF SARCOMERIC THIN FILAMENT PROTEINS

The thin filament proteins that principally comprise the cardiac sarcomere are actin, tropomyosin, and the troponin T, I, and C complex. There are 2 striated muscle actins (skeletal and cardiac) which are highly conserved at the protein level. In striated muscle, actin is the protein that principally interacts with myosin to regulate muscle contraction and relaxation. The ATP-hydrolyzing activity of myosin transduces chemical energy into mechanical movement which is dramatically accelerated in the presence of actin. The tropomyosin-troponin complex which is bound to actin inhibits the actin-myosin reaction; this inhibition is reversed by an increase of calcium (Ca^2+^) binding to troponin C. In cardiac myofilaments, 80% of the striated muscle actin is the cardiac isoform and 20% is skeletal muscle actin isoform.

**Cardiac actin** genes often encode missense mutations that lead to HCM. However, 2 mutations are linked to heritable forms of DCM: R312H and E361G. These mutations occur in the immobilized actin end which is attached to the Z band and intercalated disc^47,48^. Ablation of the cardiac actin gene leads to severe structural and functional defects; most of these homozygous actin-ablation mice do not survive to birth, and the remainder die within the first 2 weeks postpartum^49^. However, these mice can be rescued by ectopic expression in the heart with enteric smooth muscle actin, but the hearts of these mice exhibit an enlarged, hypertrophic phenotype with reduced contractility. To date, there do not appear to be any mouse models of DCM with amino acid mutations in the cardiac actin gene.

**Tropomyosin** is a coiled-coil dimer that plays an essential role in the regulation of contraction/relaxation of striated and smooth muscle. There are 4 tropomyosin (TPM) genes: TPM1, TPM2, TPM3, and TPM4 (α, β, γ, and δ, respectively) which all display a very high degree of conservation among species. The human heart contains 90 – 94% α-Tpm protein levels, 3 – 5% β-Tpm isoform, and 3% α-Tpmk isoform^50^. In the adult mouse heart, α-Tpm expression is 98%, with 2% β-Tpm expression^51^.

At least 11 distinct mutations have been found in TPM1 that lead to DCM. To investigate the cellular, molecular, and physiological processes associated with the development of this cardiomyopathic process, several mouse models were generated that demonstrate a DCM phenotype^21^. We engineered the first mouse model of a sarcomeric thin filament protein that leads to DCM through the substitution of a lysine for a glutamic acid at amino acid 54 (E54K)^24^. This is a mutation in TPM1 that occurs in humans and leads to DCM. It should be noted that there is a 100% amino acid identity between human and mouse Tpm so the genetically-engineered mutations in our transgenic mice mimic those found in human patients. Histological and morphological analyses of these hearts revealed development of DCM with progression to heart failure and death often ensuing by 6 months. Decreases in left ventricular fractional shortening and impaired systolic and diastolic function was evident, along with decreased sensitivity to Ca^2+^ and decreased tension generation in cardiac myofilaments.

Structural analyses found a decrease in Tpm flexibility with the E54K amino acid change; this alteration in flexibility influences Tpm’s binding to actin. In collaboration with R. J. Solaro, we found that these DCM E54K hearts exhibited a significant decrease in their Tpm phosphorylation which indicated that altered phosphorylation may be a factor in the linkage of the E54K mutation to DCM^52^. As presented below, additional work by our group found that alterations in Tpm phosphorylation do cause a DCM hypertrophic phenotype^53,54^.

The usage of *in vitro* biochemical studies is another approach that is used to understand the structural and physiological effects of DCM mutations. These studies often use purified or recombinant sarcomeric proteins (i.e. actin, myosin, Tpm) to assay protein-protein properties and interactions, such as motility and stiffness, to determine the functional consequences of the mutations under investigation. Various investigators have employed these biophysical analyses to examine hypertrophic cardiomyopathic and DCM mutations found in thin filament proteins^55–58^. In an examination of the Tpm mutations M8R, K15N, and A277V that are associated with the development of DCM, studies were conducted using these *in vitro* systems. The M8R and K16N mutations destabilized the interaction between the N- and C-termini of Tpm in the overlap junction region and reduced the Tpm affinity for actin^55^. These changes can possibly lead to a reduction in the regulation of cooperativity that may contribute to the DCM phenotype.

Our investigations revealed the adult human heart expresses 3 Tpm striated muscle isoforms: α-Tpm, β-Tpm, and α-Tpmk^50^. We also found that the quantitative level of a cardiac-specific α-Tpmk isoform is increased in human patients with DCM and heart failure^50^. We generated transgenic mice that express this unique isoform in the heart. Increased levels of α-Tpmk were incorporated into the myofilaments and lead to a DCM cardiomyopathic phenotype, coupled with systolic and diastolic dysfunction and decreased myofilament Ca^2+^ sensitivity. Biophysical work demonstrated less structural stability and weaker actin-binding affinity of α-Tpmk compared with wildtype α-Tpm protein, thus providing a plausible mechanism for the consequences of the Tpm isoform switch observed in DCM and heart failure patients.

Previous studies in our laboratory demonstrated that during embryonic and fetal cardiogenesis, the murine heart expressed both α-Tpm and β-Tpm isoforms; α-Tpm is the predominant isoform in the adult heart with 98% protein expression^51^. To address whether β-Tpm could substitute for α-Tpm, we generated mice that overexpressed the β-Tpm isoform in the heart^59^. Moderate expression of β-Tpm resulted in no morphological or physiological alterations; however, high expression (80% β-Tpm) lead to a severe DCM phenotype with death occurring within 14 days postpartum^60^. There is significant chamber dilation, thrombus formation in both atria and ventricles, along with diastolic dysfunction. In a follow-up investigation, we found that treating these high expression β-Tpm mice with cyclosporin, an inhibitor of calcineurin, can rescue these mice from the cardiomyopathic phenotype^61^. This work demonstrates that inhibitors of calcineurin may play a potential therapeutic role in the treatment of heart disease.

Phosphorylation of cardiac proteins can play a major role in the regulation of the physiological performance of the heart. Phosphorylation of thin filament proteins dramatically affect systolic and diastolic function. To address how Tpm phosphorylation affects cardiac function, we generated transgenic mice that express a phosphorylation mimetic at the sole phosphorylation site: S283D^54^. Our results show that high expression of the Tpm S283D transgene leads to a severe dilated cardiomyopathic phenotype resulting in death within 1 month of birth. Moderate expression of the transgene causes a mild myocyte hypertrophy and fibrosis, along with diastolic dysfunction but without affecting lifespan.

**Troponin T** plays an essential role in the sarcomere by linking the troponin complex to tropomyosin. Troponin (TnT) acts by modulating the availability of actin to the myosin head via the movement of Tpm. There are 3 TnT genes: cardiac (TNNT2), slow (TNNT1), and fast (TNNT3) that produce multiple striated muscle TnT mRNA and protein isoforms. These isoforms play non-redundant roles in contraction of different muscle types.

There have been 13 DCM-inducing mutations linked to TNNT2^62^. The first reported mutation causing DCM was a deletion of K210 in the cardiac TNNT2 gene^63^. A common mechanism associated with this cardiac pathology in most TnT DCM mutations is a decrease in myofilament Ca^2+^ sensitivity, which is similar to mutations in Tpm that cause DCM. This disruption of TnT-Tpm interactions impairs the systolic/diastolic processes which triggers the DCM phenotype.

Animal models have been developed to analyze TnT mutations that lead to DCM. The deletion of lysine at amino acid 210 (del210K) in TnT can affect both young and older patients.^22^ This deletion mutation was engineered in a knock-in mouse where it resulted in DCM with reduced survival.^65^ Cardiac muscle fibers showed a decreased Ca^2+^ sensitivity for force generation, with markedly enlarged hearts leading to heart failure and frequently sudden death. Interestingly, a positive inotropic agent, pimobendane, which increases myofilament Ca^2+^ sensitivity, had profound effects of preventing cardiac hypertrophy, heart failure and sudden death^65^. Additional studies demonstrated that the R141W mutation in TnT, along with most other TnT mutations that cause DCM, exhibit a decreased sensitivity to Ca^2+^ in myofibers^65,66^.

**Troponin I** is a subunit of troponin that is responsible for inhibition of actomyosin ATPase activity. In the absence of Ca^2+^, cardiac Tnl (cTnl) inhibits contraction through its interactions with Tpm and actin; this inhibition is relieved during muscle contraction upon Ca^2+^ binding to cTnC. Only 4 mutations in the cTnl gene have been reported to lead to DCM. One of these mutations, A2V, exhibits an autosomal recessive mode of inheritance. Two of the mutations (K35Q, N185K) are autosomal dominant, and the inheritance of the fourth mutation (P16T) has not been reported^62,67^. All of these DCM mutations in cTnl decrease the maximum activity and myofilament Ca^2+^ sensitivity of actin-myosin S1 ATPase. They also reduce the binding affinity of the regulatory site of cTnC protein in the thick filament. There have been no reported mouse models with mutations in cTnl that lead to the cardiomyopathic DCM phenotype.

**Troponin C** is a key regulatory protein in striated muscle contraction where its function is to bind Ca^2+^ and trigger actomyosin interactions to initiate sarcomeric contraction. The troponin C (TnC) gene expresses TnC in both cardiac and slow skeletal muscle isoforms. Six TnC mutations have been reported that lead to DCM: Y5H, Q50R, E59D-D75Y, M103I, I148V, and E159D^68^. Currently, no DCM-inducing loss-of-function alleles have been detected in the TnC N-lobe, possibly because missense mutations in this region cause a lethal phenotype^69^. Interestingly, some of the DCM mutations in TnC do not always exhibit a decreased myofilament Ca^2+^ sensitivity, and sometimes have no effect on this property^62,68^. A DCM transgenic mouse has been developed that harbors the I61Q TnC mutation. Isolated myofilaments from the hearts of these mice have a reduced myofilament tension, reduced cardiac function, and increased diastolic left ventricular chamber size with a decreased septal wall thickness^70^.

**Premature ventricular contractions** are usually regarded as benign in the absence of structural heart disease; however, they are increasingly recognized as an important potentiator in the development of DCM. Recently, a swine model of premature ventricular contractions (PVC) which evolves into many features of DCM was developed^71,72^. A ventricular pace-maker was implanted into the hearts which delivers a 50% PVC burden with pacing for 14 weeks. Results show a PVC-induced cardiomyopathy develops with a leftward shift in the unipolar voltage distribution and increased interstitial fibrosis. These symptoms are similar to findings in humans with PVC-induced DCM cardiomyopathy. A similar PVC model developed in canines resulted in a DCM phenotype characterized by a reduced left ventricular ejection fraction, left ventricular dilation, and eccentric hypertrophy^73^. Also, there was an increase in left ventricular systolic and diastolic dimensions while decreasing wall thickness and increasing fibrosis. More recent results with this canine model show a hypertrophic response in cardiomyocytes, but little increase in the calcineurin/NFAT hypertrophic activation markers^74^. However, there was an upregulation of the AKT/mTOR and MARK pathways suggesting that a molecular program is functioning to induce an adaptive remodeling response to the frequent PVCs. Whether this adaptive hypertrophy can compensate for the pathological response with other additional treatments remains to be seen.

## Conclusions

This review focuses on many of the leading causes of DCM and its pathological structural and physiological alterations. Both genetic and non-genetic causes are operative that can lead to this cardiomyopathic disease. In addition to gene modifying systems that have been implemented to generate DCM animal models, physiological manipulations have also been utilized to mimic and study this disease. Through these *in vivo* and *in vitro* model systems, significant advances in our knowledge have occurred. For example, consistent physiological findings across many of these models are that left ventricular systolic and diastolic dysfunction, with reduced blood ejection fraction often occurs. Cardiomyocyte hypertrophy is usually observed, as are dysrhythmias, myocarditis, and fibrosis. In many cases, DCM leads to heart failure, but this is not inevitable, especially in cases of non-familial causes of DCM, such as drug related incidence. By further experimentation with DCM animal models, it is hoped that improved therapeutic agents and remedies can be developed, with the eventual application for usage in humans.
